# From straw to salmon: a technical design and energy balance for production of yeast oil for fish feed from wheat straw

**DOI:** 10.1186/s13068-023-02392-2

**Published:** 2023-09-20

**Authors:** Christian Sigtryggsson, Hanna Karlsson Potter, Volkmar Passoth, Per-Anders Hansson

**Affiliations:** 1 Department of Energy and Technology, Swedish University of Agricultural Sciences, 750 07 Uppsala, Sweden; 2 Department of Molecular Sciences, Swedish University of Agricultural Sciences, 750 07 Uppsala, Sweden

**Keywords:** Lignocellulose, Biochemical conversion, Oleaginous yeast, Primary energy demand, Microbial oil, Biorefinery

## Abstract

**Background:**

Aquaculture is a major user of plant-derived feed ingredients, such as vegetable oil. Production of vegetable oil and protein is generally more energy-intensive than production of the marine ingredients they replace, so increasing inclusion of vegetable ingredients increases the energy demand of the feed. Microbial oils, such as yeast oil made by fermentation of lignocellulosic hydrolysate, have been proposed as a complement to plant oils, but energy assessments of microbial oil production are needed. This study presents a mass and energy balance for a biorefinery producing yeast oil through conversion of wheat straw hydrolysate, with co-production of biomethane and power.

**Results:**

The results showed that 1 tonne of yeast oil (37 GJ) would require 9.2 tonnes of straw, 14.7 GJ in fossil primary energy demand, 14.6 GJ of process electricity and 13.3 GJ of process heat, while 21.5 GJ of biomethane (430 kg) and 6 GJ of excess power would be generated simultaneously. By applying economic allocation, the fossil primary energy demand was estimated to 11.9 GJ per tonne oil.

**Conclusions:**

Fossil primary energy demand for yeast oil in the four scenarios studied was estimated to be 10–38% lower than for the commonly used rapeseed oil and process energy demand could be met by parallel combustion of lignin residues. Therefore, feed oil can be produced from existing non-food biomass without causing agricultural expansion.

## Introduction

Atlantic salmon (*Salmo salar*) is one of the most commonly produced aquaculture species worldwide, with a tenfold increase in production volumes in the past three decades [1]. With a global increase in salmon production and fed aquaculture in general, competition for feed resources with other livestock sectors is expected to increase [2]. The composition of salmon feed has changed considerably since the 1990s, with fish meal and fish oil gradually being replaced by cheaper plant-derived proteins and lipids, such as soy protein concentrate, wheat gluten and rapeseed oil [3–5]. While replacing marine ingredients with vegetable substitutes would ease the pressure on marine ecosystems, some plant ingredients have been shown to be more resource- and energy-intensive than the fish oil and fish meal they replace [6, 7]. To sustain a growing aquaculture industry, alternative feed resources based on waste streams and currently under-utilised resources need to be developed [8].

Microorganisms have been identified as promising candidates for producing lipids (single-cell oil) with a fatty acid composition similar to vegetable oil (VOs) [9–13], (Table 1). However, the fatty acid composition varies to some extent depending on species and growth phase. Ascomycetous yeasts, such as *Lipomyces starkeyi*, have a fatty acid profile similar to more saturated VOs such as palm or olive oil with 36–41% saturated fatty acids (SAT), 58–59% monounsaturated fatty acids (MUFA) and 0–5% polyunsaturated fatty acids (PUFA) in total fatty acids. The basidiomycetous yeast *Rhodotorula toruloides* has a SAT proportion of 30%, MUFA 53% and PUFA 16% [14], and is thus more similar to VOs of the MUFA class [15], such as rapeseed oil. The proportion of linolenic acid (18:3) can reach similar levels as in rapeseed or soybean oil (Table 1). However, the PUFA in yeast and vegetable oil include linoleic and linolenic acid (18:2 and 18:3, respectively), but no eicosapentaenoic acid [EPA, 20:5(*n*-3)] or docosahexanoic acid [DHA, 22:6(*n*-3)]. Microbial oil, like VO, mainly supplies metabolic energy and can to some extent provide the precursor (linolenic acid) to synthesis of EPA and DHA. However, salmon, like human and other animals, are inefficient in converting linolenic acid into EPA and DHA, which must still be added to the diet of the fish [16].

**Table 1:** Fatty acid profile of some oleaginous yeasts and vegetable oils

**Yeast species**	Palmitic (16:0)	Stearic (18:0)	Oleic (18:1)	Linoleic (18:2)	Linolenic (18:3)
*Lipomyces starkeyi* [12, 17]	27–36	1–9	35–51	2–19	0–4
*Rhodotorula toruloides* [13, 18]	13–28	2–13	39–56	12–26	2–8
*Rhodotorula babjevae* [12]	10–16	2–5	23–63	7–17	3–18
Vegetable oil [15]					
Palm	43.8	4.4	39.1	10.2	0.3
Olive	12.1	2.6	72.5	9.4	0.6
Rapeseed	5.1	1.7	60.1	21.5	9.9
Soybean	10.8	3.9	23.9	52.1	7.8

Fat-synthesising yeasts and other fungi were actually used for oil production over a century ago, following urgent fat shortages in Germany during the First World War [19, 20]. Since then, several genera of oleaginous yeast have been evaluated, amongst them *Rhodotorula*, comprising numerous fat-forming species accumulating lipids in concentrations of up to 70% by mass [21]. Several attempts have been made to industrialise and commercialise production of microbial fats, including γ-linolenic acid-rich oil [22] and a cocoa-butter equivalent [23]. Despite being technically successful, production in those cases was limited by high costs for fermentation and substrates. In the past decade, lignocellulosic hydrolysate has been suggested as a low-cost substrate for future oleaginous biorefineries [24–27].

Harvest residues from the agricultural sector have been suggested as a suitable resource and feedstock for production of various bio-oils, second-generation biofuels and chemicals [28, 29]. Every year, 730 Mt of rice straw, 350 Mt of wheat straw and 200 Mt of maize stover are produced globally, with the vast majority left in the field [30, 31]. When using straw as a feedstock for microbial conversion into yeast oil, the polysaccharides of interest (cellulose and hemicellulose) first need to be hydrolysed into monosaccharides. Physicochemical pre-treatments such as steam explosion followed by enzymatic hydrolysis make pentose and hexose sugars [32] accessible for most oleaginous yeasts to assimilate [27].

Microbial utilisation of straw for feed purposes is not a new idea [33], although methods and techniques have evolved and have been refined. In a laboratory study by Blomqvist et al. [34], oleaginous yeast fed with straw hydrolysate from wheat was found to form lipids that were a successful replacement for vegetable oil in the feed of the salmonid Arctic char (*Salvelinus alpinus*). The performance in terms of energy and resource use of a scaled-up version of that system in which agricultural residues are utilised for salmon feed production was examined in the present study. The analysis was based on a biorefinery plant fermenting straw hydrolysate to lipids that fully replace the current vegetable oil component (20% by mass) in salmon feed. The biorefinery was assumed to fully utilise residual streams and by-products that arise throughout the process, to better meet the high internal energy demand and increase the competitiveness of production. A technical design and a mass and energy balance at systems level—from straw to salmon—were developed.

## Methods

### System overview

A system analysis was performed, where the system outcomes considered were mass and energy balance. The mass balance was represented by the yield of farmed salmon, yeast oil and other valuable outputs per tonne of straw input. The energy balance covered the fossil primary energy demand (PED_fossil_) per tonne of salmon, per tonne of yeast oil produced and per tonne of wheat straw used, which was the sum of all non-renewable primary energy used for inputs in the manufacturing process. Construction of the biorefinery plant itself was not considered. Mass and energy balance modelling was carried out in Aspen Plus, using a similar design to that presented by [35] except for the biodiesel upgrading step. Assumptions made regarding fermentation conditions and yields were partly based on work by Blomqvist et al. [28] and Brandenburg et al. [14, 34] and recent unpublished laboratory results by those authors. In the case of co-production of two or more products, economic allocation was used to determine the contribution of each product to the total energy demand and the impact of the allocation method chosen was evaluated in a sensitivity analysis.

**Fig. 1:**
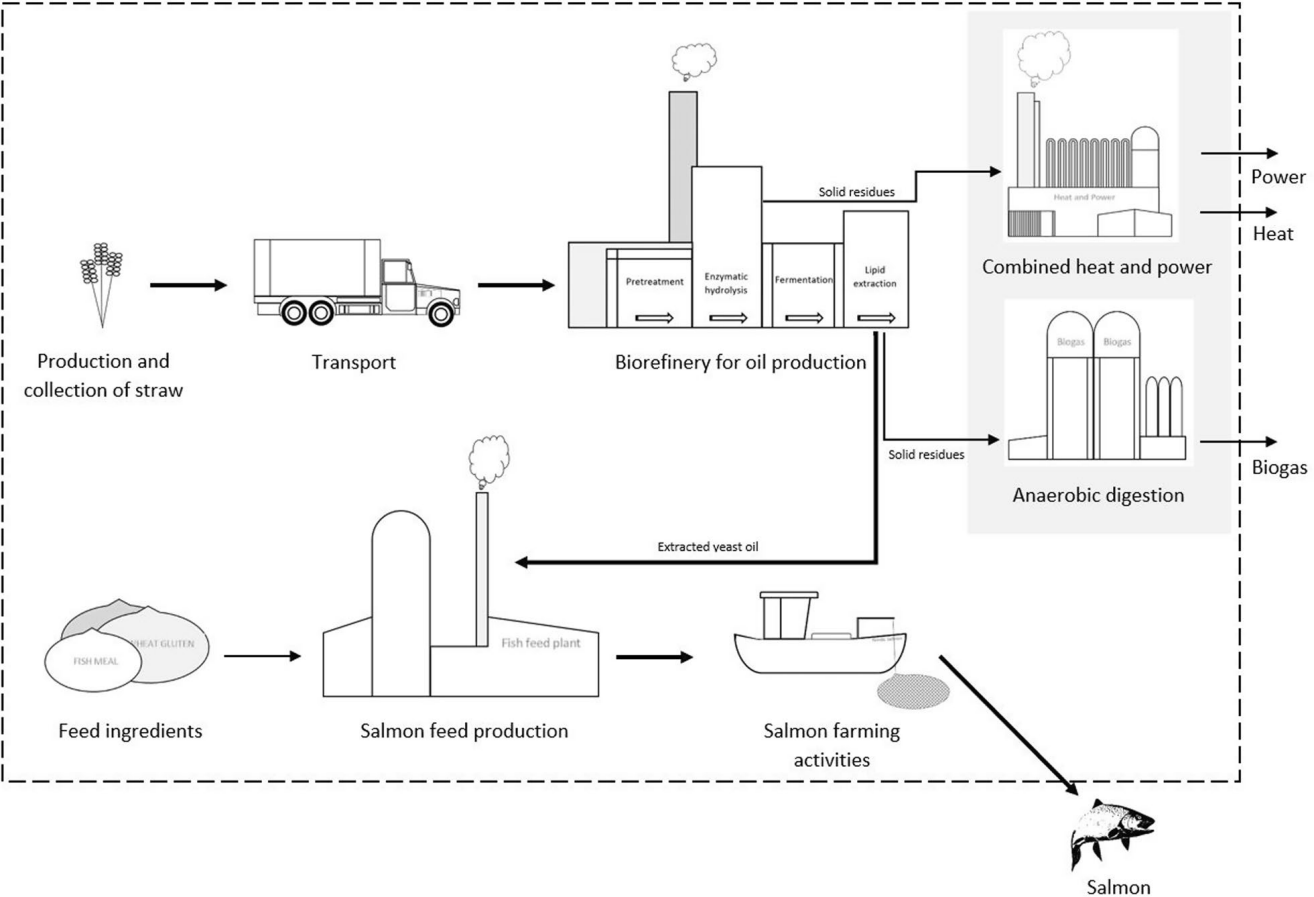
System overview of the main process steps from field production of straw to salmon farming and parallel utilisation of process residues for generation of power and heat

The system studied is schematically presented in Fig. 1, where the dotted line represents the system boundaries. The system starts with wheat straw production and harvest, including all relevant inputs, such as fertilisers, diesel and pesticides, followed by transport of straw to the biorefinery by truck. In the biorefinery, the cellulose and hemicellulose are hydrolysed and fermented into lipid-rich yeast biomass. The lipids are extracted and added to the salmon feed pellets, replacing all vegetable oil in the fish feed factory. The remaining yeast biomass is anaerobically digested into biomethane, while the solid residues (lignin) from hydrolysis are utilised in a combined heat and power plant. The feed pellets are distributed to Norwegian salmon farms and the system assessed ends with 1 tonne of fresh salmon (Fig. 1).

### Fish feed production and fish farming system

Norwegian feed data and farming conditions were used for this study, together with primary energy demand values previously determined for Norwegian salmon feed and farming by Boissy et al. [36]. However, salmon feed composition has changed considerably in the past decade, with increasing inclusion of plant-derived ingredients, and a more recent and representative picture of Norwegian salmon feed provided by Aas et al. [4] was also used as a reference feed mix in this study (see Table 2). Primary Energy Demand values found in Ecoinvent [37], the French database ECOALIM [38], Broekema and Blonk [39] and Skontorp Hognes [40] were used to calculate PED_fossil_.

**Table 2:** Composition (% by mass) of ingredients in Norwegian salmon feed and fossil primary energy demand (PED_fossil_) per tonne of ingredient

Feed ingredient	Inclusion rate, %	PED_fossil_ (MJ/tonne ingredient)	References
Wheat	8.9	5733	[37]
Other carbohydrate sources	1.8	5733^a^	[37]
Soy protein concentrate	19	9335	[39]
Fish meal	14.5	9979	[37]
Wheat gluten	9	35,280	[39]
Faba beans	4	2779	[38]
Maize gluten	3.4	23,240	[38]
Pea protein concentration	1.3	10,800	[40]
Sunflower meal	1.1	8780	[40]
Sunflower protein	0.5	8780^b^	[40]
Other vegetable proteins	2.3	9335^c^	[40]
Rapeseed oil	20.1	13,365	[38]
Fish oil	10.4	19,714	[37]
Micronutrients	3.9	32,549	[38]

^a^Assumed to be wheat

^b^Assumed to be sunflower meal

^c^Assumed to be soy protein concentrate

Fish oil and vegetable oil are currently included in fish feed by vacuum coating, a method, where the oil is absorbed by pre-extruded and dried feed pellets [41]. From a technical perspective, this makes it possible to modify the mix of oils added to the feed without changing the production line or energy requirements, which in turn allows for use of alternative lipid sources. Yeast lipids from two oleaginous yeasts, *Lipomyces starkeyi* and *Rhodotorula toruloides*, grown on wheat hydrolysate and included in the feed of Arctic char, have recently been evaluated in two studies [34, 42]. The fatty acid composition of the feed was found to be comparable to that of the ordinary feed, as were all growth and health parameters of the fish fed with yeast-based feed. In the present study, we assumed full replacement of current rapeseed oil (20.1% by mass) with yeast oil in the base scenario.

### Biorefinery and production of yeast oil from lignocellulosic biomass

According to the International Energy Agency, “Biorefinery is the sustainable processing of biomass into a spectrum of marketable products (food, feed, materials, chemicals) and energy (fuels, power, heat)” [43]. The conversion can be either thermochemical (gasification and pyrolysis) or biochemical (involving pre-treatment followed by fermentation) [44], which was the focus of this study. Using oleaginous microorganisms (bacteria, fungi) in fermentation, lipid-based chemicals can also be synthesised [45]. A biorefinery producing biodiesel and biomethane from wheat straw was described and evaluated previously by Karlsson et al. [35]. Production of the yeast oil assessed in this study started with production and harvesting of wheat straw. As straw is a byproduct of grain production, PED_fossil_ was determined using economic allocation to be 270 MJ eq/tonne straw [37]. The biorefinery was designed to process 84,100 tonnes of straw (dry mass) per year, producing about 9200 tonnes of yeast oil and was assumed to be located in southern Sweden, in an area with good straw supply within 45 km transport distance [46]. The energy demand for transportation was 66 MJ/tonne straw (dry mass) including empty return [47].

At the plant, the straw was assumed to be fed into a grinder and chopped to 6 mm size, with a total energy demand of 60 MJ/tonne straw [48]. The chemical composition of the straw was assumed to be: glucan 36%, lignin 26%, xylan 20%, ash 5%, arabinan 3%, protein 3%, extractives 3%, acetate 2% and galactan 1% [49]. The ground straw was assumed to be soaked in dilute sulphuric acid (corresponding to 0.22 tonne 18 M H_2_SO_4_/tonne straw) and exposed to steam explosion (190 °C, 10 min) before being subjected to enzymatic hydrolysis. Separate hydrolysis and fermentation were assumed, meaning that enzymatic treatment was followed by solid/liquid separation, where only the liquid hydrolysate was transferred to the fermenter, in a process carried out according to considerations and assumptions made by Karlsson et al. [35]. The enzyme used was Cellic® 1.0 from Novozymes A/S, with a primary energy demand of 13 MJ/kg enzyme (Jesper Kløverpris, Novozymes A/S, personal communication 19 April 2016), in a dose corresponding to 13.5 kg enzyme/tonne straw. Total sugar recovery from pre-treatment and enzymatic hydrolysis was assumed to be 619 g/kg straw.

The yeast *Rhodotorula babjevae* was chosen for fermentation, due to its ability to accumulate high concentrations of lipids and its fast growth. The yeast was first assumed to be propagated in the presence of liquid ammonia (approximately 10 kg N/tonne straw) in a separate tank for 48 h and then pumped to the main fermenter, where the yeast was continuously fed with hydrolysate for 72 h in nitrogen-limited conditions to promote lipid accumulation, with lipid yield set to 0.20 g/g sugar. Agitation of the propagator tank and fermenter tank was assumed to require 2.2 MJ of electric energy per m^3^ liquid and aeration was set to 1 vvm (volume unit of air per unit liquid and minute) provided by a compressor (85% isentropic efficiency). Due to the presence of acetic acid in the hydrolysate, the pH had to be kept close to neutral to avoid inhibition of yeast metabolism [17]. This pH does not inhibit growth of potential contaminants, so incoming air was assumed to be passed through a filter to prevent contamination. Although the yeast can accumulate up to 65% lipids per dry cell biomass [14], the lipid content was set to 50% (dry weight) after 72 h in the model for the base scenario. For extracting the oil, the yeast cells were assumed to be mechanically disrupted by a homogeniser and mixed stepwise with hexane to dissolve the lipids. The yeast biomass was then separated from the lipid-solvent phase and hexane was recovered by evaporation, with an assumed loss of 0.5% [50]. The yeast oil was assumed to have similar technical properties to rapeseed oil (the energy content was assumed to be 37 MJ/kg) and, therefore, did not need any further refinement before being used for feed purposes.

The solid residues remaining from enzymatic hydrolysis (mainly lignin) were assumed to be diverted to a combined heat and power plant and the solid residues from fermentation (yeast biomass) to a biogas reactor. These processes were modelled as described by Karlsson et al. [35]. The remaining digestate was assumed to be used as biofertiliser, while the process water was treated and recirculated within the plant.

### Scenario analysis

Besides the base scenario described above, the following scenarios were analysed:

- Fermentation + 1 day (FERM + 1), with the lipid proportion of cell dry weight reaching 65% after 4 days.
- Fermentation − 1 day (FERM − 1), with the fermentation time shortened by 1 day, resulting in a lipid proportion of 40%.
- NITROREC, with recirculation of nitrogen in the outgoing process water back to yeast propagation.

All three scenarios reflect possible changes in biorefinery design that could be highly relevant for reducing process energy demand and PED_fossil_ per tonne of oil produced. The FERM + 1 and FERM − 1 scenarios are both supported by the lipid production curve described by Brandenburg et al. [14] and by findings by Karlsson et al. [35] that fermentation is the most energy-consuming step in the biorefinery. Fermentation can either be stopped earlier, with a lower lipid yield related to total sugar concentration, or prolonged to maximise lipid yield, with both scenarios having a substantial impact on process energy demand. The liquid ammonia used for yeast propagation before lipid accumulation has a high PED_fossil_. After disruption and solvent extraction, some of the nitrogen in the yeast can be recovered and returned to the yeast propagation tank, thereby lowering the PED_fossil_ of the yeast oil. Scenario NITROREC assumed full recovery of free ammonia dissolved in the process water.

## Results

### Energy demand for salmon feed and farming

The PED_fossil_ value for the current salmon feed mix, determined using the inclusion percentage and PED_fossil_ data for the respective ingredients listed in Table 2, was estimated to be 14.4 GJ per tonne (Table 3). The amount of feed required to produce one unit of salmon, based on the economic feed conversion ratio (weight of feed fed/weight of salmon harvested), is reported to be 1.30 [4]. This means that the feed required for production of 1 tonne of Norwegian salmon had a PED_fossil_ value of 18.7 GJ. As previously stated, production of plant proteins and oils can be highly energy-intensive (see Table 3), with two of the main fish meal and fish oil substitutes (wheat gluten and rapeseed oil, respectively) making up more than 40% of the total PED_fossil_ of the feed mix. Based on the work of Boissy et al. [36], PED_fossil_ for feed manufacturing and for farming activities on Norwegian salmon farms (including transport of feed) was estimated to be 3300 MJ and 6800 MJ per tonne of fish produced, respectively. Thus, the total PED_fossil_ for 1 tonne of salmon, including feed ingredients, feed production, transportation of feed and farming was 28.8 GJ.

**Table 3:** Fossil primary energy demand (PED_fossil_, MJ) of the current salmon feed mix and percentage contribution of respective ingredients to PED_fossil_

Current feed mix	PED_fossil_ (MJ)	Contribution (%) to PED_fossil_ of current feed mix
Wheat	510	4
Other carbohydrate sources	100	1
Soy protein concentrate	1770	12
Fish meal	1450	10
Wheat gluten	3180	22
Faba beans	110	1
Maize gluten	790	5
Pea protein concentration	140	1
Sunflower meal	100	1
Sunflower protein	40	0.3
Other vegetable proteins	220	1
Rapeseed oil	2690	19
Fish oil	2050	14
Micronutrients	1270	9
Total (GJ)	14.4	100

### Energy demand of the biorefinery

#### Primary energy demand of inputs

**Fig. 2:**
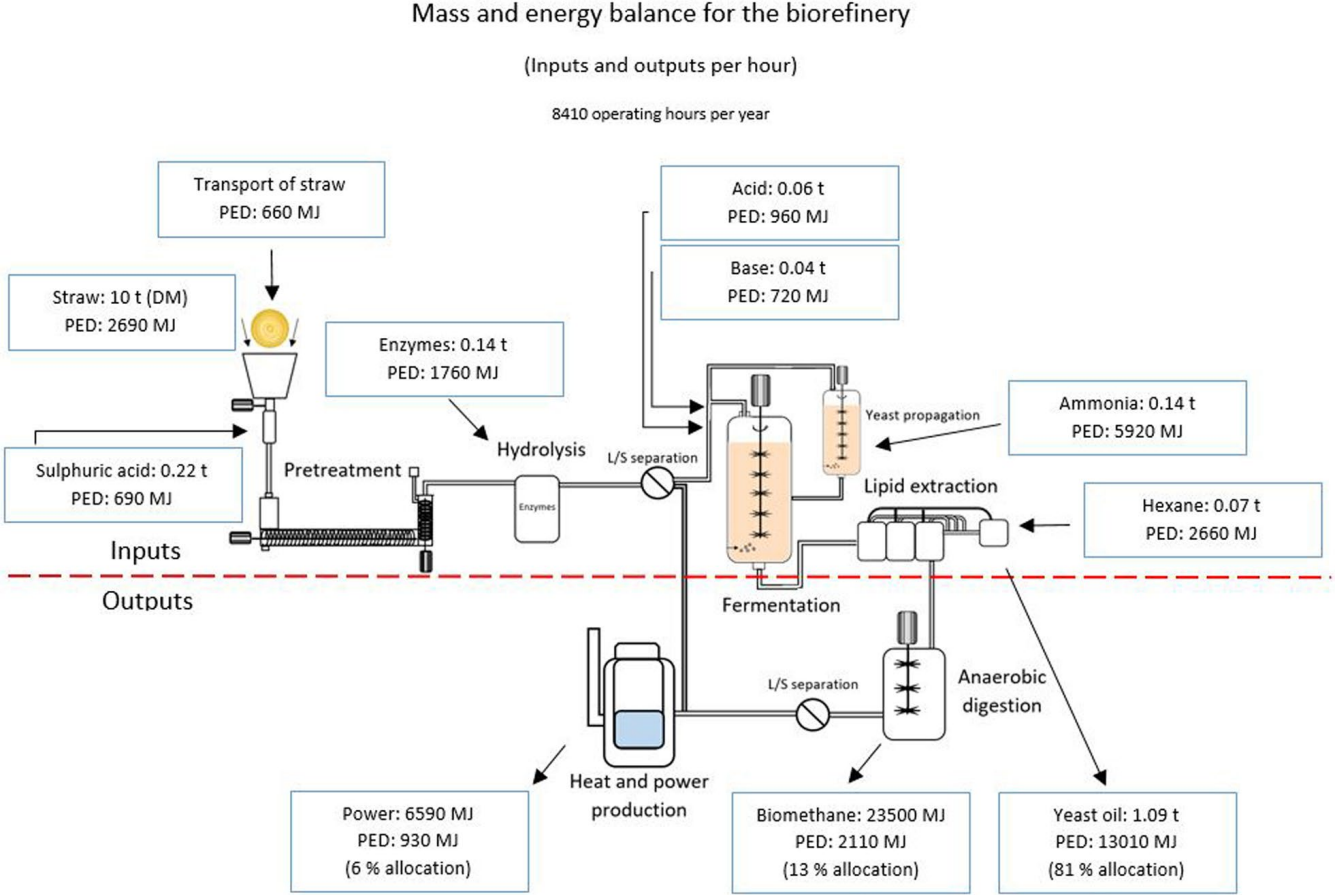
Mass and energy balance for lipid, biomethane and power production in the biorefinery in the base scenario. Mass and energy are presented for each input and output in tonnes (t) or MJ per hour. The energy demand for biomethane, power and yeast oil was determined by applying economic allocation. Primary energy demand (PED) data for inputs except for transport and enzymes were taken from the Ecoinvent database [37]

Full replacement of vegetable oil in the feed required to produce 1 tonne of salmon meant that 261 kg of yeast oil (inclusion rate of oil × economic feed conversion ratio) had to be produced in the biorefinery. Figure 2 shows the biorefinery and its respective inputs and outputs separated by a red dotted line, with the mass in tonnes and primary energy demand in MJ for each input and output per hour presented in blue boxes. The total PED_fossil_ of the inputs was estimated to be 16 GJ. Mineral nitrogen, hexane and enzymes were the most energy-intensive chemical inputs in the refinery, making up 65% of total PED_fossil_. The process energy for the plant (heat and power) was covered by combustion of lignin in the solid straw residues, so fossil primary energy used for heating and powering the equipment was already included in PED_fossil_ of the straw.

Per hour, 1.09 tonnes of yeast oil, 23.5 GJ (0.47 t) of biomethane and 6.6 GJ (1.8 MWh) of power (net) were produced in the biorefinery (Fig. 2). The oil-producing capacity of the biorefinery was sufficient to meet the oil demand for about 5.42 tonnes of feed, or 4.17 tonnes of salmon, per hour. The power output was excess electricity remaining when the internal power demand of the biorefinery had been met. Economic allocation was applied to estimate the contribution of the respective products to total PED_fossil_. The price of electricity (northern Europe) was set to $11.51 per GJ ($1 = 0.81 Euro) and the value of the biomethane was assumed to equal that of European natural gas ($7.32 per GJ) [51, 52]. As there is currently no commercial trade in yeast oil, it was assumed to be competitively priced relative to rapeseed oil ($972 per tonne). Accordingly, the yeast oil was given an allocation factor of 0.81, corresponding to 11.9 GJ per tonne oil. All prices were a 10-year average for 2010–2020 and a sensitivity analysis was performed to analyse the sensitivity to price and the allocation method chosen (3.3).

The PED_fossil_ of the yeast oil (11.9 GJ/tonne oil) was found to be lower than for rapeseed oil (13.4 GJ/tonne), resulting in PED_fossil_ of 14.1 GJ per tonne of feed on assuming full substitution of vegetable oil (approx. 14.4 GJ/tonne for reference feed mix). Including the energy demand for feed production and fish farming, salmon fed with yeast oil-based feed would accordingly have a PED_fossil_ of 28.5 GJ per tonne of fish, compared with 28.8 GJ for salmon fed the reference mix. Although the yeast oil itself has a lower PED_fossil_, it is too small to make a significant difference per tonne of salmon.

#### Process energy demand

Although the yeast oil had relatively low PED_fossil_, a substantial amount of process energy was used in the biorefinery. Table 4 shows the PED_fossil_ of inputs and the heat and electricity demand for each sub-process in the factory per tonne of straw input and yeast oil produced. Per tonne of yeast oil, a total of 12 GJ electricity and an additional 22.3 GJ heat were required to run the biorefinery. However, as some of the heat could be recovered by heat exchangers and reused in the plant, the net heat demand was reduced by more than half, to 10.8 GJ. The most heat-demanding sub-process was found to be straw pre-treatment, steam explosion in particular (76% of the total), while the most power-demanding sub-processes were stirring the fermenters and running the air compressors during lipid accumulation (75% of the total).

**Table 4:** Energy demand of inputs, process heat and electricity per tonne of straw (dry matter (DM) basis) and per tonne of oil (allocated), and mass balance for biomethane and yeast oil production in the base scenario (not allocated)

Inputs	Per tonne DM straw	Per tonne of oil produced (allocated)	Units
Straw			
Straw farming	270	2000	MJ
Transport	66	490	MJ
Pre-treatment			
Electricity	73	540	MJ
Heat	2270	16,910	MJ
Sulphuric acid	69	510	MJ
Hydrolysis			
Electricity	33	250	MJ
Heat	0	0	MJ
Enzymes	176	1310	MJ
Fermentation			
Electricity	1200	9000	MJ
Heat	0	0	MJ
Ammonia	590	4400	MJ
Acid and base for pH adjustment	170	1240	MJ
Lipid extraction			
Electricity	130	950	MJ
Heat	620	4630	MJ
Hexane	270	1970	MJ
Anaerobic digestion			
Electricity	150	1150	MJ
Heat	98	730	MJ
Wastewater treatment			
Electricity	18	130	MJ
Heat	0	0	MJ
Total electricity demand	1610	11,960	MJ
Total heat demand	2990	22,270	MJ
Total heat demand with recovery	1450	10,780	MJ
Primary energy demand	1.6	11.9	GJ

Outputs		Not allocated	
Yeast oil	109	1	kg
Gross electricity	2270	20,810	MJ
Gross heat	3290	30,180	MJ
Biomethane	2350	21,500	MJ
Excess electricity	660	6030	MJ
Excess heat	1840	16,880	MJ

The electricity and heat demand was fully met by production from the biorefinery’s combined heat and power plant, where the remaining solid residues (less than 1/3 of straw input) were combusted. Gross heat and electricity production per tonne of yeast oil was 30.2 GJ and 20.8 GJ, respectively, which means that 6 GJ of excess electricity was sent to the grid. In addition, 21.5 GJ (0.43 tonnes) of biomethane were produced per tonne of oil.

### Scenario and sensitivity analysis

Since fermentation is the most power-intensive process in the biorefinery and the effect remains constant over time, two of the scenarios (FERM + 1 and FERM − 1) tested the effects of changing the fermentation time by ± 1 day on power consumption. By extending the fermentation to 4 days in scenario FERM + 1, more yeast oil (+ 30%), but less biomethane (− 23%) and excess electricity (− 30%), were produced compared with the base scenario (Table 5). Decreasing the fermentation time to 2 days (FERM − 1) resulted in a 20% reduction in yeast oil production, but a 17% and 34% increase in production of biomethane and excess electricity, respectively. In the NITROREC scenario, where ammonia in the process water was reused for yeast propagation, the mineral nitrogen input was 84% lower, leading to the lowest PED_fossil_ per tonne of oil of all scenarios (Table 5). However, the effects on the feed mix were marginal because of the relatively low inclusion rate of oil and PED_fossil_ per tonne of salmon varied between 27.5 and 28.5 GJ.

**Table 5:** Mass and energy balance for the base scenario and the three alternative scenarios

	Units	Base case	FERM + 1	FERM − 1	NITROREC
Products					
Yeast oil	kg/t straw	109	142	87	109
Biomethane	MJ/t straw	2350	1800	2750	2350
Excess electricity	MJ/t straw	660	460	880	660
Inputs					
Enzymes	MJ/t straw	176	176	176	176
Hexane	MJ/t straw	270	240	290	270
Ammonia	MJ/t straw	590	590	590	100
PED_fossil_					
Yeast oil	GJ/t oil	11.9	9.8	12	8.3
Fish feed	GJ/t feed	14.1	13.7	14.1	13.4
1 tonne salmon	GJ/t fish	28.5	27.9	28.5	27.5

The impact of variations in the price of yeast oil, biomethane and power was tested in a sensitivity analysis by increasing one price at a time by 10% (Table 6). The effect on the fossil energy footprint of the yeast oil was in general marginal, with a 10% increase in the price of yeast oil increasing its PED_fossil_ by less than 2%. The outcomes of economic allocation were also compared with allocation based on energy content. The result showed that using energy allocation had a substantial effect on the outcome, reducing PED_fossil_ of the yeast oil by almost 30%.

**Table 6:** Results of sensitivity analysis in which the price of each commodity was increased by 10%. Applying allocation based on energy instead of economic allocation when estimating PED_fossil_ of the yeast oil was also analysed

	Yeast oil + 10%	Biomethane + 10%	Electricity + 10%	Energy allocation
PED_fossil_ yeast oil	+ 1.76%	− 1.30%	− 0.58%	− 29.4%

## Discussion

The PED_fossil_ value of the yeast oil in the base scenario (11.9 GJ/tonne) was lower than PED_fossil_ values for rapeseed oil and other vegetable oils reported in the literature (Table 2). The ECOALIM value for rapeseed oil (presented in Table 2) is 13.4/tonne, while Boissy et al. [36] estimated PED_fossil_ of rapeseed oil and palm oil to be 26.8 and 17.4 GJ/tonne, respectively. Even without applying an allocation factor, and thus letting the yeast oil bear the full primary energy cost of the biorefinery, it appeared to be competitive with other plant oils regarding fossil-based energy demand. As process energy was supplied by the biorefinery’s integrated heat and power plant, with only a minor contribution to PED_fossil_, minimising the amount of external input energy required for mineral N, solvent and enzymes used in the process appears to be of key relevance for achieving low fossil-based energy demand. Scenario analysis showed that substantial reductions can be achieved, e.g., returning mineral nitrogen to fermentation could alone decrease PED_fossil_ of the oil by about 30% (Table 5).

Norway is the world’s leading salmon producer, with a market share of 50%, and the salmon industry alone is responsible for more than 1% of global rapeseed oil consumption. In a hypothetical scenario with all vegetable oil in Norwegian salmon feed replaced with yeast oil, a total of 3.7 Mt straw (0.83% DM) would be required in the base scenario with 109 kg oil produced per tonne of straw, corresponding to 34 times the capacity of the biorefinery described in this paper. On implementing the more energy-demanding FERM + 1 scenario, with higher oil production, the corresponding straw input could be decreased to 2.8 Mt straw. With estimated global wheat straw production of 350 Mt annually [31], there would be more than enough feedstock for supplying the global salmon industry with oil. However, as transport of straw needs to be minimised to maintain an overall favourable energy balance (90 MJ increase in PED_fossil_ per tonne for every 10 km transport distance added), straw-fed biorefineries would need to be located in arable areas with good availability of straw and low competition from livestock production.

Stirring the yeast broth during yeast propagation and lipid accumulation was the most power-demanding process in the biorefinery. There are many aspects to consider when optimising yeast oil production from an energy use point of view, depending on whether the aim is to save fossil energy or overall energy, or to maximise oil production. As lipid accumulation levels off on days 3–4, there is no incentive from a process energy point of view for continuing fermentation once lipid yield per unit of electricity consumed stops increasing. On the other hand, a vast amount of fossil energy has already been invested by the time the inputs are used in the biorefinery, so our results indicate that an additional fermentation day is favourable in terms of PED_fossil_ per tonne of yeast oil.

Energy analysis showed that the biorefinery used 12 GJ of electricity and 10.8 GJ of heat (including recovery) per tonne of yeast oil (37 GJ). As an example, it would take 3940 TJ of electricity and 3540 TJ of heat to produce enough yeast oil to replace the 328,000 tonnes of vegetable oil used annually in the Norwegian salmon industry. The biorefinery considered in this study was assumed to be powered in a similar way to many Nordic pulp and paper mills [53], using solid residues for producing steam and electricity. Although the energy used for this was non-fossil, the amount was considerable in terms of general efforts by society to save energy. Alternative uses could be to produce green power for the grid or other even more valuable products from the lignin [54]. Our scenario analysis demonstrated it is possible to better balance the total outputs of the biorefinery to the inputs of available straw, chemicals and process energy. The scenario where fermentation time was shortened by 1 day (FERM − 1) increased PED_fossil_ by 95 MJ per tonne of oil compared with the base scenario, but saved 2 GJ of electricity per tonne of oil.

When interpreting the results, it is important to understand the impact of the economic allocation method. For reflecting each product’s share of the total energy burden, economic allocation was deemed to be the better choice, since allocation by mass or by energy content would not be fully applicable or relevant as the products differ in their economic significance. The prices of rapeseed oil, natural gas and electricity have fluctuated significantly in recent years, not least in the post-pandemic period. The sensitivity analysis showed a marginal effect on the PED_fossil_ of yeast oil of a moderate price increase (10%), while even doubling the reference price of rapeseed oil did not increase the estimated PED_fossil_ of the yeast oil by more than 10%. Applying energy allocation, which would reduce the estimated energy footprint of the oil by 30% in comparison, would be better justified if the oil were refined to biofuel. However, although the allocation basis may theoretically change the estimated energy demand of the products, it does not change the fact that the overall energy burden of the biorefinery is a direct consequence of yeast oil production.

Enzymes, solvents and mineral nitrogen were the most energy-demanding external inputs used in the biorefinery. As shown in scenario NITROREC, there can be substantial energy-saving potential in returning nitrogen in the yeast cells to the fermentation tank. In the same way, reducing the need for hexane by changing the extraction method or simply avoiding separation of lipids from biomass could decrease PED_fossil_ even further. Use of supercritical carbon dioxide (sCO_2_) has been suggested as a relatively cheap, easy and safe alternative to lipid extraction that also would enable separation of other valuable components, such as carotenoids [55]. Although sCO_2_ has been assessed for its economic and environmental impacts by Taher et al. [56] and partly for its energy demand by Monari et al. [57], the overall impact on PED_fossil_ for the extraction phase when applying sCO_2_ separation remains to be determined. Including the whole yeast without prior extraction, as in the laboratory study by Blomqvist et al. [34], would take away the need for any chemical solvent and, as whole yeast contains proteins, it could also replace some of the protein in the feed mix and thereby lower the estimated PED_fossil_ even further. However, that would require complete redesign of fish pellet production, as the current process cannot handle a mix of wet ingredients and fat. Moreover, components of yeast cells may have unforeseen effects on the fish. Studies have shown that growth and health parameters of the fish are largely not negatively affected [34, 42], but enhanced liver weight has been observed in Arctic char fed a diet containing *R. toruloides* [42]. This indicates that further and longer studies are required to ultimately determine the optimal proportion of yeast biomass in fish feed.

Besides wheat straw, other lignocellulosic biomass types could be used for yeast oil production, including other straw types but also wood. Using forest residues such as branches and tops in regions with good availability would increase the amount of biomass available for yeast oil production, and ongoing laboratory studies by our research groups and others show promising results for *Rhodotorula* spp. fed with wood hydrolysate ([58], unpublished results). Although yeast oil was shown to perform better than ordinary vegetable oil in terms of PED_fossil_ in this study, there is a risk that this production method, if implemented on a large scale, soon would face similar sustainability issues as the crop production it replaced. Harvest residues of any kind are likely to be regarded as valuable feedstock in the future and demand for biomass is thus likely to increase, so it is reasonable to assume that biorefineries would focus on producing more profitable products to be competitive in terms of feedstock supply. Co-production of carotenoids and beta-glucan with the yeast oil, although not covered in this study, can be important in achieving this. For the salmon industry, nutritious feed is crucial to maintaining healthy salmon populations [59]. An oil that could supply both energy and desirable micro-components to the feed mix would make the yeast oil technology even more attractive.

## Conclusions

The fossil primary energy demand (PED_fossil_) of producing one tonne (37 GJ) of yeast oil, 21.5 GJ of biomethane and 6 GJ of excess power from straw in the biorefinery studied was determined to be 14.7 GJ, while the process energy demand for its production was 14.6 GJ of power and 13.3 GJ of heat. The oil was assumed to replace rapeseed oil in salmon feed. According to the results obtained, yeast oil could be produced with lower PED_fossil_ than rapeseed oil, despite energy-intensive processing steps in the biorefinery. When using harvest residues currently left in the field as feedstock, production of this alternative feed oil would not contribute to land use changes to the same degree as continued expansion of agriculture. However, the analysis revealed trade-offs between process energy demand, oil yield and the amount of feedstock used in the process that would have to be considered if scaling up the biorefinery. Previous studies have shown that yeast oil can be successfully included in the feed of salmonids, so its potential as a future vegetable oil substitute is likely to depend on production economics and feedstock availability. Further studies are required to determine the extent to which yeast oil can replace VO in fish feed.

## Data Availability

All data generated or analysed during this study are included in this published article.

## References

[1] Iversen A, Asche F, Hermansen Ø, Nystøyl R (2020). Production cost and competitiveness in major salmon farming countries 2003–2018. Aquaculture.

[2] Tacon AGJ, Metian M (2015). Feed matters: satisfying the feed demand of aquaculture. Rev Fish Sci Aquacult.

[3] Ytrestøyl T, Aas TS, Åsgård T (2015). Utilisation of feed resources in production of Atlantic salmon (*Salmo salar*) in Norway. Aquaculture.

[4] Aas TS, Ytrestøyl T, Åsgård T (2019). Utilization of feed resources in the production of Atlantic salmon (*Salmo salar*) in Norway: an update for 2016. Aquaculture Reports.

[5] Poblete EG, Drakeford BM, Ferreira FH, Barraza MG, Failler P (2019). The impact of trade and markets on Chilean Atlantic salmon farming. Aquacult Int.

[6] Pelletier N, Tyedmers P, Sonesson U, Scholz A, Ziegler F, Flysjo A (2009). Not all salmon are created equal: life cycle assessment (LCA) of global salmon farming systems. Environ Sci Technol.

[7] Newton RW, Little DC (2018). Mapping the impacts of farmed Scottish salmon from a life cycle perspective. Int J Life Cycle Assess.

[8] Albrektsen S, Kortet R, Skov PV, Ytteborg E, Gitlesen S, Kleinegris D (2022). Future feed resources in sustainable salmonid production: a review. Rev Aquacult.

[9] Ratledge C (1993). Single cell oils—have they a biotechnological future?. Trends Biotechnol.

[10] Ochsenreither K, Glück C, Stressler T, Fischer L, Syldatk C (2016). Production strategies and applications of microbial single cell oils. Front Microbiol.

[11] Bharathiraja B, Sridharan S, Sowmya V, Yuvaraj D, Praveenkumar R (2017). Microbial oil—a plausible alternate resource for food and fuel application. Biores Technol.

[12] Brandenburg J, Poppele I, Blomqvist J, Puke M, Pickova J, Sandgren M (2018). Bioethanol and lipid production from the enzymatic hydrolysate of wheat straw after furfural extraction. Appl Microbiol Biotechnol.

[13] Chmielarz M, Blomqvist J, Sampels S, Sandgren M, Passoth V (2021). Microbial lipid production from crude glycerol and hemicellulosic hydrolysate with oleaginous yeasts. Biotechnol Biofuels.

[14] Brandenburg J, Blomqvist J, Shapaval V, Kohler A, Sampels S, Sandgren M (2021). Oleaginous yeasts respond differently to carbon sources present in lignocellulose hydrolysate. Biotechnol Biofuels.

[15] Dubois V, Breton S, Linder M, Fanni J, Parmentier M (2007). Fatty acid profiles of 80 vegetable oils with regard to their nutritional potential. Eur J Lipid Sci Technol.

[16] Sprague M, Dick JR, Tocher DR (2016). Impact of sustainable feeds on omega-3 long-chain fatty acid levels in farmed Atlantic salmon, 2006–2015. Sci Rep.

[17] Brandenburg J, Blomqvist J, Pickova J, Bonturi N, Sandgren M, Passoth V (2016). Lipid production from hemicellulose with *Lipomyces starkeyi* in a pH regulated fed-batch cultivation: lipid production from hemicellulose with *Lipomyces starkeyi*. Yeast.

[18] Nagaraj YN, Burkina V, Okmane L, Blomqvist J, Rapoport A, Sandgren M (2022). Identification, quantification and kinetic study of carotenoids and lipids in R*hodotorula toruloides* CBS 14 cultivated on wheat straw hydrolysate. Fermentation.

[19] Schmidt E (1947). Eiweiß- und Fettgewinnung über Hefe aus Sulfitablauge. Angew Chem.

[20] Lundin H (1950). fat synthesis by micro-organisms and its possible applications in industry. J Inst Brew.

[21] Nigam PS, Singh A (2014). Fermentation (industrial)|production of oils and fatty acids. Encyclopedia of food microbiology.

[22] Ratledge C, Akoh C (2005). Microbial production of gamma-linolenic acid. Handbook of functional lipids. functional foods and nutraceuticals.

[23] Ratledge C, Tan KH (1990). Oils and fats: production, degradation and utilization by yeasts. Yeast—biotechnology and biocatalysis.

[24] Huang C, Chen XF, Xiong L, de Chen X, Ma LL, Chen Y (2013). Single cell oil production from low-cost substrates: the possibility and potential of its industrialization. Biotechnol Adv.

[25] Mast B, Zöhrens N, Schmidl F, Hernandez R, French WT, Merkt N (2014). Lipid production for microbial biodiesel by the oleagenious yeast *Rhodotorula glutinis* using hydrolysates of wheat straw and miscanthus as carbon sources. Waste Biomass Valor.

[26] Patel A, Arora N, Sartaj K, Pruthi V, Pruthi PA (2016). Sustainable biodiesel production from oleaginous yeasts utilizing hydrolysates of various non-edible lignocellulosic biomasses. Renew Sustain Energy Rev.

[27] Passoth V, Sandgren M (2019). Biofuel production from straw hydrolysates: current achievements and perspectives. Appl Microbiol Biotechnol.

[28] Saini JK, Saini R, Tewari L (2015). Lignocellulosic agriculture wastes as biomass feedstocks for second-generation bioethanol production: concepts and recent developments. 3 Biotech.

[29] Smil V (1999). Crop residues: agriculture’s largest harvest. Bioscience.

[30] Kim S, Dale BE (2004). Global potential bioethanol production from wasted crops and crop residues. Biomass Bioenerg.

[31] Sarkar N, Ghosh SK, Bannerjee S, Aikat K (2012). Bioethanol production from agricultural wastes: an overview. Renew Energy.

[32] Galbe M, Wallberg O (2019). Pretreatment for biorefineries: a review of common methods for efficient utilisation of lignocellulosic materials. Biotechnol Biofuels.

[33] Han YW (1978). Microbial utilization of straw (a review). Advances in applied microbiology.

[34] Blomqvist J, Pickova J, Tilami SK, Sampels S, Mikkelsen N, Brandenburg J (2018). Oleaginous yeast as a component in fish feed. Sci Rep.

[35] Karlsson H, Ahlgren S, Sandgren M, Passoth V, Wallberg O, Hansson PA (2016). A systems analysis of biodiesel production from wheat straw using oleaginous yeast: process design, mass and energy balances. Biotechnol Biofuels.

[36] Boissy J, Aubin J, Drissi A, van der Werf HMG, Bell GJ, Kaushik SJ (2011). Environmental impacts of plant-based salmonid diets at feed and farm scales. Aquaculture.

[37] EcoinventCenter (2022). Ecoinvent data v3.9 ecoinvent reports no. 1–25.

[38] Wilfart A, Espagnol S, Dauguet S, Tailleur A, Gac A, Garcia-Launay F (2016). ECOALIM: a dataset of environmental impacts of feed ingredients used in French animal production. PLoS ONE.

[39] Broekema R, Blonk H (2009). Milieukundige vergelijking van vleesvervangers.

[40] Skontorp Hognes E, Ziegler F, Sund V (2011). Carbon footprint and area use of farmed Norwegian salmon.

[41] Chaabani A, Labonne L, Tercero CA, Picard JP, Advenier C, Durrieu V (2020). Optimization of vacuum coating conditions to improve oil retention in Trout feed. Aquacult Eng.

[42] Brunel M, Burkina V, Pickova J, Sampels S, Moazzami AA (2022). Oleaginous yeast *Rhodotorula toruloides* biomass effect on the metabolism of Arctic char (*Salvelinus alpinus*). Front Mol Biosci.

[43] IEA. Annual Report 2020. IEA Bioenergy; 2020. https://www.ieabioenergy.com/wp-content/uploads/2021/04/IEAB-Annual-Report-2020.pdf.

[44] Wertz JL, Bédué O (2013). Lignocellulosic biorefineries.

[45] Jin M, Slininger PJ, Dien BS, Waghmode S, Moser BR, Orjuela A (2015). Microbial lipid-based lignocellulosic biorefinery: feasibility and challenges. Trends Biotechnol.

[46] Ekman A, Wallberg O, Joelsson E, Börjesson P (2013). Possibilities for sustainable biorefineries based on agricultural residues—a case study of potential straw-based ethanol production in Sweden. Appl Energy.

[47] Karlsson H, Börjesson P, Hansson PA, Ahlgren S (2014). Ethanol production in biorefineries using lignocellulosic feedstock—GHG performance, energy balance and implications of life cycle calculation methodology. J Clean Prod.

[48] Miao Z, Grift TE, Hansen AC, Ting KC (2011). Energy requirement for comminution of biomass in relation to particle physical properties. Ind Crops Prod.

[49] Linde M, Jakobsson E, Galbe M, Zacchi G (2008). Steam pretreatment of dilute H2SO4-impregnated wheat straw and SSF with low yeast and enzyme loadings for bioethanol production. Biomass Bioenerg.

[50] Davis R, Kinchin C, Markham J, Tan E, Laurens L, Sexton D, et al. Process design and economics for the conversion of algal biomass to biofuels: algal biomass fractionation to lipid- and carbohydrate-derived fuel products. 2014. p. NREL/TP-5100-62368, 1159351. Report No.: NREL/TP-5100-62368, 1159351. http://www.osti.gov/servlets/purl/1159351/. Accessed 11 Oct 2022.

[51] The World Bank. Commodity markets, Pink Sheet Data, monthly prices; 2023. https://thedocs.worldbank.org/en/doc/5d903e848db1d1b83e0ec8f744e55570-0350012021/related/CMO-Historical-Data-Monthly.xlsx.

[52] Nord Pool. Market Data, System price; 2023. https://www.nordpoolgroup.com/en/Market-data1/Dayahead/Area-Prices/SYS1/Yearly/?view=table.

[53] Lipiäinen S, Kuparinen K, Sermyagina E, Vakkilainen E (2022). Pulp and paper industry in energy transition: Towards energy-efficient and low carbon operation in Finland and Sweden. Sustain Prod Consum.

[54] Glasser WG (2019). About making lignin great again—some lessons from the past. Front Chem.

[55] Sahena F, Zaidul ISM, Jinap S, Karim AA, Abbas KA, Norulaini NAN (2009). Application of supercritical CO2 in lipid extraction—a review. J Food Eng.

[56] Taher H, Giwa A, Abusabiekeh H, Al-Zuhair S (2020). Biodiesel production from *Nannochloropsis gaditana* using supercritical CO2 for lipid extraction and immobilized lipase transesterification: economic and environmental impact assessments. Fuel Process Technol.

[57] Monari C, Righi S, Olsen SI (2016). Greenhouse gas emissions and energy balance of biodiesel production from microalgae cultivated in photobioreactors in Denmark: a life-cycle modeling. J Clean Prod.

[58] Passoth V, Brandenburg J, Chmielarz M, Martín-Hernández GC, Nagaraj Y, Müller B (2023). Oleaginous yeasts for biochemicals, biofuels and food from lignocellulose-hydrolysate and crude glycerol. Yeast.

[59] Sahlmann C, Djordjevic B, Lagos L, Mydland LT, Morales-Lange B, Øvrum Hansen J (2019). Yeast as a protein source during smoltification of Atlantic salmon (*Salmo salar* L.), enhances performance and modulates health. Aquaculture.

